# Molecular pathogenesis of the schwannomatosis genes and genetic testing strategies

**DOI:** 10.1007/s10689-025-00507-2

**Published:** 2025-11-24

**Authors:** Miriam J. Smith

**Affiliations:** 1 Manchester Centre for Genomic Medicine, St Mary’s Hospital, Manchester University Hospitals NHS Foundation Trust, Manchester Academic Health Sciences Centre (MAHSC), Manchester, UK; 2 Division of Evolution, Infection and Genomics, School of Biological Sciences, Faculty of Biology, Medicine and Health, University of Manchester, Manchester, UK

**Keywords:** Schwannomatosis, *NF2*, *LZTR1*, *SMARCB1*, Genetic testing, Mosacism, Molecular pathogenesis

## Abstract

The three major schwannomatosis genes, *NF2*, *LZTR1* and *SMARCB1*, are all located within approximately 9 megabases on chromosome 22 and cause three genetically distinct conditions with significant clinical phenotypic overlap. All forms of schwannomatosis predispose to the development of multiple schwannomas, but display differences in tumour location and long-term prognosis. In addition, high levels of mosaic disease can complicate clinical diagnosis. Genetic diagnosis can be critical for distinguishing between the three conditions to optimise clinical management, especially in cases of mosaic disease. This review summarises the distinctions between the clinical and genetic characteristics of each form of schwannomatosis and discusses the genetic analytic tools that are typically used to detect the variants found in these conditions.

## Introduction

The schwannomatoses are a group of genetically distinct autosomal dominant tumour predisposition syndromes with overlapping clinical presentations [1]. The three main genes associated with schwannomatosis are *NF2*, *SMARCB1* and *LZTR1*. All forms of schwannomatosis (SWN) predispose to the development of multiple nerve sheath tumours that may occur throughout the nervous system, on cranial, spinal and/or peripheral nerves. *NF2*-related schwannomatosis (*NF2*-SWN) is the most common form (with a prevalence of 1 in 60,000 according to UK data [2]) and is characterised by a predisposition to multiple schwannomas, meningiomas and ependymomas. Almost everyone with non-mosaic *NF2*-SWN will develop bilateral vestibular schwannomas, which confer a high risk of profound hearing loss requiring hearing aids or auditory brainstem implants. Over 50% of people with *NF2*-SWN will develop at least one meningioma and approximately 30% will develop an ependymoma. Meningiomas are a marker of disease severity due to their location-dependent morbidity. *NF2*-SWN also confers characteristic ocular features including retinal hamartoma, lenticular opacities and epiretinal membranes.

The non-*NF2*-related schwannomatoses normally manifest multiple non-intradermal peripheral schwannomas, and spinal schwannomas [3]. These tumours are frequently associated with a substantial amount of pain that does not respond to medication, and it has been suggested that pain may differ depending on the predisposing gene i.e. *SMARCB1* or *LZTR1* [4, 5]. Although painful schwannomas have generally been shown to express higher levels of inflammatory cytokines, such as interleukin-6, interleukin-8 and vascular endothelial growth factor (VEGF) than non-painful schwannomas, the secretomes of schwannomas associated with a pathogenic *SMARCB1* variant differ from those with a pathogenic *LZTR1* variant [5, 6].

Non-vestibular cranial schwannomas are less common than peripheral schwannomas. Meningiomas have not been reported in *LZTR1*-SWN, but have been observed in approximately 5% of people with *SMARCB1*-SWN [3, 7]. In contrast, vestibular schwannomas have not been definitively associated with *SMARCB1*-SWN, but have been observed in approximately 5% of *LZTR1*-SWN [8].

Each form of schwannomatosis has a different prognosis, e.g. a reduced life expectancy in *NF2*-SWN. They also have different clinical management requirements e.g. Bevacizumab therapy for *NF2*-SWN [9] and pain medication or surgery for painful schwannomas in non-*NF2*-SWN. Accurate genetic diagnosis is critical to distinguish between these different conditions, for provision of accurate assessment of genetic risk for individuals who may have mosaic disease, and for optimal clinical management and monitoring. It has been demonstrated that in the *NF2*-SWN population there is a 6% 20-year risk of malignancy following radiation therapy for benign tumours in comparison to < 1% risk for tumours that have not undergone irradiation, so radiotherapy is not recommended as a first line treatment for people with a germline pathogenic *NF2* variant [10].

The importance of genetic testing is highlighted by the inclusion of genetic diagnosis as a criterion to distinguish between each form of schwannomatosis in current clinical diagnostic guidelines [1]. Here, the distinctions between these conditions are summarized and the genetic analytic tools that are typically used to detect the variants found in these conditions are discussed.

## Genetic characteristics of *NF2*-SWN

**Fig. 1:**
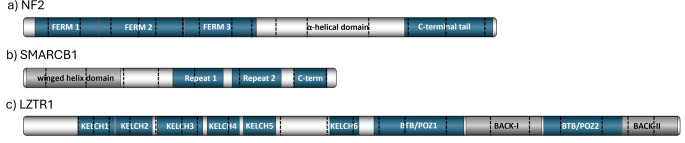
Schematic diagrams of the schwannomatosis gene products, indicating which exons encode each feature. a) NF2 contains three N-terminal FERM domains, which fold into a cloverleaf conformation. This is followed by an alpha-helical domain and a C-terminal tail. b) SMARCB1 has an N-terminal winged helix domain, two repeat domains and a C-terminal domain, which interacts with the nucleosome acidic patch. c) LZTR1 has six N-terminal KELCH domains, which fold into a beta-propellor. There are two BTB/POZ domains towards the C-terminus and two partial BACK domains, which may facilitate substrate recognition

*NF2*-SWN was initially thought to constitute a sub-form of neurofibromatosis. However, neurofibromas are not a part of the clinical phenotype [1]. The discovery of the *NF2* gene in 1993 [11, 12], definitively distinguished *NF2*-SWN as a separate disorder from neurofibromatosis. The *NF2* gene is 95kb in size and is located on chromosome 22q12.2. It contains 17 coding exons and produces two major isoforms which differ by their final exon. The matched annotation from NCBI and EMBL_EBI (MANE) select transcript is isoform 1 (NM_000268.4) which includes exons 1–15 and 17, encoding a 595 amino acid protein, and it is this isoform that is considered to be clinically relevant [13]. Isoform 2 (NM_016418.5) includes all exons. However, exon 16 contains a termination codon, which precludes translation of exon 17, and results in translation of a shorter, 590 amino acid, protein with an alternate C-terminus [13]. Figure 1 shows schematic diagrams of the main protein domains for each of the schwannomatosis gene products and indicates which exons encode each feature.

The *NF2* protein product, moesin, ezrin, radixin-like protein (Merlin), acts as a link between the actin cytoskeleton and components of the cell membrane. As such, Merlin contains three FERM (4.1, ezrin, radixin, moesin) domains, which are involved in protein–protein interactions [14]. FERM domain 1 is encoded by the first 3 exons, FERM domain 2 is encoded by exons 4–6, and FERM domain 3 is encoded roughly by exons 7–9. These three domains fold into a ‘cloverleaf’ conformation [15]. These are followed by an α-helical domain, encoded by exons 10–13, and a C-terminal tail domain encoded by exons 14–15. The alpha-helical domain folds to bring the C-terminal and N-terminal domains together and when found in this folded ‘closed’ conformation, Merlin is unable to bind actin. When the N- and C-terminal domains dissociate Merlin forms an ‘open’ conformation, and forms homodimers with altered protein binding characteristics [13, 16–18]. These features highlight the importance of Merlin protein conformation and suggest why pathogenic variants that cause disruption of normal protein folding may lead to tumourigenesis.

As a tumour suppressor gene, the *NF2* gene requires biallelic inactivation to initiate tumour development. Screening studies of the *NF2* gene in affected individuals have found a range of germline variant types that act as a ‘first hit’, predisposing to *NF2*-SWN, including truncating (nonsense, frameshift and some splice variants), as well as non-truncating (missense and some splice variants) [19–21]. Pathogenic variants have been found throughout the coding region, except the last exons i.e. the alternatively spliced exons 16 and 17. Most germline pathogenic *NF2* variants are single nucleotide variants or small indels. Splice-region variants account for approximately 30% of pathogenic *NF2* variants, while intragenic copy number variants (CNVs), whole gene deletions and larger structural variants (SVs) account for approximately 20% of predisposing variants in families with *NF2*-SWN. Genotype–phenotype analyses for the smaller variants have shown that their location within the gene correlates with disease severity [19, 22, 23]. Variants that occur towards the beginning of the gene normally cause a more severe clinical phenotype than those that occur towards the end of the gene, indicated by markers of disease severity such as the presence of meningiomas and earlier age at onset.

A pathogenic variant located in the 5’-untranslated region (5’-UTR), c.66_65insT, has also been identified in multiple members of two *NF2*-SWN families [24]. Variants in non-coding regions tend to be more difficult to characterise, as their effects are typically more challenging to interpret. However, bioinformatic analysis of the c.66_65insT variant suggested that it alters the reading-frame of an existing upstream open reading frame (uORF), previously identified by ribosome profiling [25]. This frame-shift disrupts the uORF stop codon, causing translation of the uORF to continue through the canonical translation start codon of *NF2*, thus reducing expression of the wild-type transcript.

As mentioned above, approximately 20% of pathogenic *NF2* variants are larger intragenic deletions or duplications, or whole gene deletions [26]. Whole gene deletions typically lead to a milder clinical presentation than small truncating variants. This is in contrast to germline *NF1* whole gene deletions, which tend to produce a more severe phenotype [27]. The milder phenotype caused by *NF2* whole gene deletions is thought to be due to the lack of additional tumour suppressor genes in the deleted region and a reduced mechanism for the occurrence of second, somatic variants affecting the trans allele in tumours from people with a large deletion as their primary pathogenic variant [26]. This theory is supported by the lack of loss-of-heterozygosity (LOH) events in tumours from people with a germline whole gene deletion, whereas LOH is the most common second hit in tumours from people with a single nucleotide variant as their first genetic hit (identified in around 75% of these tumours). Occasionally, when the tumour being tested is a vestibular schwannoma, more than one second hit is identified, due to the multifocal development of this type of tumour [28].

A confounding genetic feature of the *NF2* gene is the very high frequency of mosaicism in individuals with de novo *NF2*-SWN [29]. It is thought that methylation during early embryogenesis at certain CpG dinucleotide sequences in *NF2* could be responsible for a large number of de novo mutation events, since a small number of recurrent nonsense variants occurring at CpG dinucleotides account for over half of de novo pathogenic *NF2* variants [30]. It has also been shown that this type of severe truncating variant is more likely to be seen in mosaic form than milder non-truncating variants. Non-truncating variants are more likely to be detected in an inherited heterozygous form due to a lack of symptomatic presentation when present in mosaic form [31]. Mosaic *NF2*-SWN generally confers a low risk of inherited disease to offspring, and the level of risk is likely to correlate with the variant allele frequency detected in lymphocytes.

A genetic severity scoring system was developed to classify predicted clinical severity based on the predisposing variant [32]. Within this system, non-mosaic truncating variants are classified as conferring severe disease (class 3). Splice variants involving exons 1–7, as well as large deletions that do not include the promoter or exon 1 and truncating variants in exons 14–15, and mosaic truncating variants in exons 2–13 are all classified as conferring moderate disease (class 2B). Missense variants, in-frame deletions or duplications, large deletions that include the *NF2* promoter or exon 1, splice variants in exons 8–15, truncating variants located in exon 1, and mosaic non-truncating variants in exons 2–13 are all classified as conferring mild disease (class 2A). When a patient meets the clinical diagnostic criteria, but no variant is identified in blood, they can be classified as a confirmed tissue mosaic if an identical *NF2* variant is found in two anatomically distinct tumours (class 1B); however, if only one tumour, or no tumours are available for confirmation these are classified as presumed tissue mosaics (class 1A).

A higher genetic severity score has been shown to correlate with a higher likelihood of developing bilateral vestibular schwannomas, intracranial meningiomas and spinal schwannomas [33]. However, the scoring system was also shown to be more accurate for the classification of severe disease phenotypes, than for patients in the milder or mosaic categories [34]. This was partially resolved by the inclusion of protein expression assays using patient fibroblast cells to determine the effects of particular pathogenic variants on Merlin function [34]. These features highlight the importance of molecular diagnosis and an understanding of the effects of each pathogenic variant on disease severity.

## Genetic characteristics of *SMARCB1*-SWN

The *SMARCB1* gene is 50kb, located 6 megabases centromeric of the *NF2* gene at 22q11.23. It contains 9 coding exons and produces two major isoforms, which differ by 27 nucleotides at the end of exon 2. The MANE select transcript is the longer transcript, NM_003073.5. This encodes a core subunit of the human SWI/SNF chromatin remodelling complex, which is involved in regulation of expression of approximately 5% of genes spread throughout the genome [35]. The SMARCB1 protein contains an N-terminal winged helix domain [36] encoded by exons 1–3, two repeat domains encoded within exons 5–6 and exons 6–7, and a C-terminal domain encoded within exons 8–9, which interacts with the nucleosome acidic patch to mediate chromatin remodelling [37] (Fig. 1b).

Germline pathogenic variants in *SMARCB1* are known to cause at least three different conditions: rhabdoid tumour predisposition syndrome (RTPS1)([38–40], schwannomatosis (*SMARCB1*-SWN) [41] and Coffin-Siris syndrome [42]. However, only RTPS1 and *SMARCB1*-SWN are tumour syndromes. Coffin-Siris syndrome is a developmental disorder leading to intellectual disability, distinctive facial features and hypoplastic fingers and toes—features that are not seen in RTPS1 or *SMARCB1*-SWN. *SMARCB1* variants that cause Coffin-Siris are typically missense or small in-frame indels located in the C-terminal domain (exons 8 and 9), although a few variants have been seen in earlier exons. Most variants have been associated with one condition or the other; however, there have been rare case reports of variants that have caused both schwannomatosis and Coffin-Siris in the same person[43, 44].

RTPS1 is an aggressive childhood cancer syndrome associated with a high risk of malignancy. Germline variants associated with RTPS1 tend to be loss-of-function variants that cause a complete loss of protein expression [38–40, 45–47], including a high frequency of whole gene deletions. Since there are families with an inherited *SMARCB1* variant identified in adults who have not developed rhabdoid tumours, it has also been proposed that there is a developmental window of opportunity for these variants to cause rhabdoid tumour development [48, 49]. In contrast, schwannomatosis related tumours tend to be benign. They develop later in life than RTPS1 tumours and the risk of malignancy is generally low. However, there have been reports of malignancy in *SMARCB1*-SWN and 4/75 *SMARCB1*-SWN patients in Manchester were reported to have developed a malignant peripheral nerve sheath tumour (MPNST) [50, 51]. Whole gene deletions are not seen in *SMARCB1*-SWN. Whole exon, or multiexon, deletions are extremely rare and are restricted to the last two exons. Variants predisposing to *SMARCB1*-SWN tend to be non-truncating, hypomorphic variants, predicted to cause a reduced expression and/or a dysfunctional protein. This is supported by immunohistochemical staining studies of SMARCB1 protein in schwannomas from people with familial schwannomas, which typically show a mosaic pattern of staining, indicating protein expression in some cells but not others [52]. This mosaic staining pattern is less common in sporadic schwannomatosis-associated schwannomas and uncommon in isolated schwannomas, which normally show diffuse SMARCB1 staining.

**Fig. 2:**
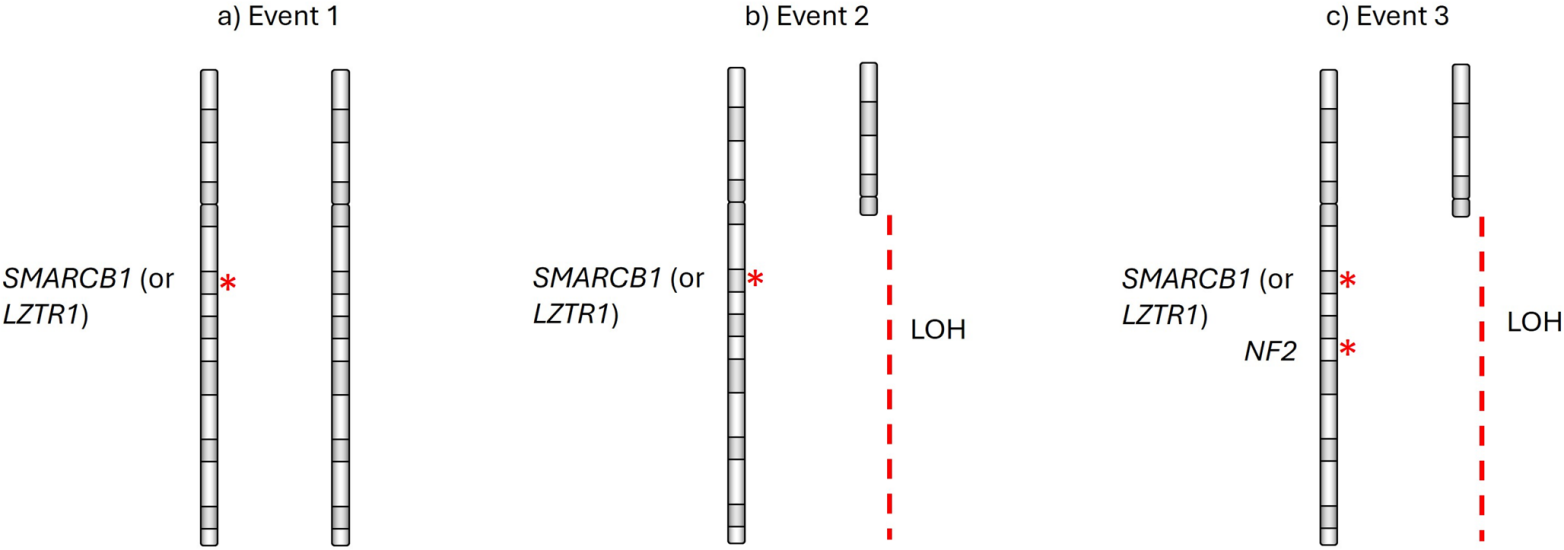
Schematic diagram showing the three-event/four-hit mechanism of gene inactivation in non-*NF2*-related schwannomatosis schwannomas. **a** germline variant as the first hit, **b** somatic loss of heterozygosity removing the remaining copy of *SMARCB1* (or *LZTR1*) and one copy of the *NF2* gene (hits 2 and 3), **c** somatic mutation of the remaining copy of *NF2* (hit 4)

Somatic pathogenic *NF2* variants are normally found in tumour DNA from non-*NF2*-SWN, which historically caused confusion in delineating other forms of schwannomatosis [53, 54]. It is now known that the mechanism of inactivation of *SMARCB1* leading to schwannoma formation follows the three-event/four-hit hypothesis [55] (outlined in Fig. 2). This begins with the initial germline variant as the first hit. This is followed by a somatic second event, involving loss of the wildtype trans allele, which removes the remaining copy of *SMARCB1*, as well as one copy of the *NF2* gene (hits 2 and 3). The third event involves somatic mutation of the remaining copy of *NF2* (hit 4). The overall effect is biallelic inactivation of both copies of *SMARCB1* and both copies of *NF2*, but with some residual expression of mutant SMARCB1 protein. All four hits are not always detectable in every non-*NF2*-related tumour, but this may be due to several reasons, such as the presence of non-tumour cell DNA in the sample.

The majority of germline *SMARCB1*-SWN variants are located towards the 5’ and 3’ end of the gene [7, 41, 49, 55, 56]. The first pathogenic variant associated with *SMARCB1*-SWN was the apparent truncating variant, c.34C > T p.(Gln12Ter), in exon 1, which was predicted to cause nonsense-mediated decay NMD [41]. However, further investigation of this variant and three other early exon 1 variants subsequently associated with *SMARCB1-*SWN and predicted to cause (c.30delC, c.38delA and c.46A > T), showed that these mutant transcripts could be rescued from degradation by reinitiation at the downstream start codon at position c.79_81 [57]. This substantiated the theory that schwannomatosis variant transcripts produce hypomorphic proteins.

The most common recurrent *SMARCB1*-SWN variant is, c.*82C > T, located in the 3’-untranslated region. This is thought to cause instability of the transcript and reduced protein expression [58]. Most unique schwannomatosis-associated variants in *SMARCB1* are missense variants, and the most common recurrent coding-region variant seen in people with schwannomatosis is the missense variant, c.41C > A p.(Pro14His), in exon 1.

*SMARCB1*-SWN-associated splice variants have been shown to cause altered transcripts that maintain the reading frame [58]. However, splice variants occurring towards the 3’ end of the gene appear to be more likely to include frame-shifting aberrations. A very deep intronic variant in intron 6, c.795 + 1498C > T, was shown to cause inclusion of a cryptic exon in intron 6, which leads to premature truncation and at least some level of nonsense-mediated decay [59]. This variant was associated with a severe schwannomatosis phenotype within the family. The reasons for this variant causing schwannomatosis, rather than RTPS1 are unclear, but it is possible that other modifier effects are responsible, or that a leaky splice effect allows for sufficient expression of wild type transcript to produce a hypomorphic effect, rather than a complete loss of protein. Two other very deep intronic variants have been found in intron 5, c.500 + 883T > G and c.500 + 887G > A, which were both found to cause splice alterations that produce transcripts with an altered reading frame [60], although the disease severity was not discussed.

## Genetic characteristics of *LZTR1*-SWN

The *LZTR1* gene is located 3 MB upstream of *SMARCB1* at 22q11.21. It spans 17kb and contains 21 exons, which produce the MANE select transcript, NM_006767.4. The leucine zipper-like transcriptional regulator 1 (LZTR1) protein product contains 840 amino acids, and forms six KELCH domains towards the N-terminus, which fold into a beta-propellor. There are two broad complex, tramtrack and brick a brac/Pox virus and zinc finger (BTB/POZ) domains towards the C-terminus. These domains appear in the opposite conformation to other leucine zipper-like family proteins, which have the KELCH domains toward the C-terminus and two BTB/POZ domains towards the N-terminus [61]. There are also two partial BTB and C-terminal kelch (BACK) domains, which may be important for facilitating substrate recognition [62] (Fig. 1c).

The BTB-KELCH superfamily of proteins are known to have roles in cell morphology, cell migration and regulating gene expression [61] and the LZTR1 protein is thought to be involved in regulating RAS ubiquitination via the cullin 3 ubiquitin ligase complex, and MAPK pathway activation [61, 63, 64].

Germline pathogenic variants in *LZTR1* are known to predispose to at least two different conditions, schwannomatosis [62] and Noonan syndrome [65]. Noonan syndrome is a developmental disorder that typically causes heart defects, delayed growth, short stature and coarse facial features, but which is not associated with an increased tumour risk. There is overlap between the variants seen in schwannomatosis and Noonan syndrome, although the variants seen in both conditions are normally inherited as autosomal dominant loss-of-function variants in *LZTR1*-SWN, while they are seen as part of a recessive inheritance pattern in Noonan syndrome. Some studies have indicated phenotypic overlap between Noonan syndrome, neurofibromatosis type 1, and schwannomatosis, including germline *LZTR1* variants associated with only café-au-lait macules [66], as well as case reports of *LZTR1*-related Noonan syndrome and vestibular schwannoma[67], or nerve tumours that are likely to be schwannomas [68]. However, the tumour risk for many variants is still unclear. A range of variant types have been observed throughout the *LZTR1* gene in people with *LZTR1*-SWN [8, 60, 62, 69–77], including nonsense, frameshift, splice alterations and missense variants. Large deletions, including whole gene deletions are extremely rare in *LZTR1*. Similar to *NF2*-SWN, this is thought to be due to a reduced mechanism for inactivation of the trans allele for people with a large deletion as the first genetic hit. This is supported by evidence from people with 22q11.2 deletion syndrome who have large germline deletions that normally include the *LZTR1* gene and appear to have a lower risk of schwannomas than the general population [78]. Since, in tumours with a large deletion as the first genetic hit, the second hit is usually a single nucleotide variant on the trans allele, this makes it less likely that a copy of the *NF2* gene on the same allele will be affected and thus, would not follow the typical three event, four hit mechanism described above. In addition, if complete inactivation of both copies of *LZTR1* did occur, it is possible that complete loss of LZTR1 protein has a different pathogenic effect compared to a potentially hypomorphic protein, or that this would be lethal to the cell.

Variant interpretation for *LZTR1* is complicated by the incomplete penetrance of *LZTR1*-SWN and the higher-than-expected frequency of some apparently pathogenic variants in the general population. Only around 50% of people from families with *LZTR1*-SWN who carry the known family pathogenic variant develop schwannomas. In addition, in gnomAD v4 (gnomAD) data, 1 in 323 people have a loss-of-function variant in *LZTR1*. Since *LZTR1*-SWN only occurs in approximately 1 in 527,000 people [2], this means that under 0.1% of people with a heterozygous loss-of-function variant in *LZTR1* actually develop schwannomatosis. This led to the current recommendation that loss-of-function variants found in people without schwannomas or a family history of schwannomas should not be used for diagnosis [79].

Studies comparing the frequency of germline loss-of-function *LZTR1*-SWN variants seen in people with schwannomatosis to loss-of-function *LZTR1* variants seen in the non-cancer gnomAD cohort [80] indicate a significantly higher frequency of *LZTR1* loss-of-function variants in schwannomatosis patients than in gnomAD data. This suggests that the association of schwannomatosis loss-of-function variants is generally robust. The strong odds ratios in case–control data for truncating variants in known tumour susceptibility genes also suggest that higher than expected frequencies of these variants in population data should not be used as conflicting evidence in variant classification [81]. However, particular care needs to be taken to assess the pathological effects and frequency of *LZTR1* variants when determining their likely pathogenicity.

Many of the missense variants associated with schwannomatosis are currently classified as variants of uncertain significance. These are particularly challenging to interpret in the absence of robust case–control data, or tumour data to confirm retention of the variant in conjunction with loss of the wildtype allele. In addition, several recurrent truncating variants have been found in people with schwannomatosis that have been seen at relatively high frequencies in UK Biobank population data e.g. c.27delG, p.Gln10Argfs*15 (101/389 101 in UK Biobank)([62, 81]. An odds ratio of 35 using case–control data indicates that this is strongly associated with schwannomatosis predisposition. Frameshifting variants associated with schwannomatosis have also been found in the last exon. Truncating variants occurring fewer than 50 nucleotides from the 3’ end of the penultimate exon are generally considered to escape nonsense-mediated decay, making them less likely to be pathogenic. However, the variants, c.2463dupA,p.(Asp822ArgfsTer29) and c.2487dupA, p.(Asp830Argfs*21), are predicted to change and extend the C-terminus of the LZTR1 protein and are considered to be pathogenic.

Segmental schwannomatosis has been seen in approximately one third of non-*NF2*-related schwannomatosis, but these cases have not generally been associated with identifiable *SMARCB1* or *LZTR1* mosaic variants [3].

## Genetic testing techniques for the schwannomatoses

The phenotypic overlap between each form of schwannomatosis makes genetic testing important for accurate diagnosis and clinical management [1, 82, 83]. Variant detection frequency in second-generation family members of *NF2*-SWN families can be as high as 95% with comprehensive genetic analysis. However, standard clinical testing normally detects approximately 87% [82]. Variant detection frequency is lower for de novo *NF2*-SWN, in which a non-mosaic pathogenic *NF2* variant can be detected in approximately 37% [29], although mosaic *NF2* variants can be detected in a further 22% and the predicted frequency of mosaic *NF2*-SWN is up to 60% for de novo disease. The frequency of detection of pathogenic non-*NF2*-SWN variants is lower than for *NF2*-SWN at around 70–86% in familial cases and 30–40% in de novo cases. In Manchester data 10.7% of 75 predictive tests on children of parents with a mosaic variant visible in blood were positive, while none of 85 predictive tests on children of affected parents with a presumed mosaic variant, but which was not visible in blood, were positive [29].

People meeting clinical diagnostic criteria for *NF2*-SWN may be tested for variants in the *NF2* gene alone in the first instance. Current targeted panel testing for people with schwannomas normally includes sequencing of *NF2*, *SMARCB1* and *LZTR1*. In some institutions, screening also includes the genes *DGCR8* and *SMARCA4*, although associations with schwannomatosis are extremely rare [84, 85]. For people with only meningiomas, germline testing may also include *SMARCE1* and *SUFU*, which are rare causes of predisposition to non-*NF2*-related meningiomas [86–88].

Targeted sequencing panels, such as Agilent SureSelect custom panels, are designed to include a minimum of the coding region of each gene and 15 nucleotides of intronic sequence at either end of each exon. Any known intronic likely pathogenic/pathogenic variants may also be tested. Single nucleotide variants and small indels can be reliably detected by this method. Targeted NGS screening does not typically identify balanced translocations or complex inversions. In addition, variants identified by these methods are typically still validated using an orthogonal method, such as Sanger sequencing.

Traditional Sanger sequencing strategies can detect germline variants down to a mosaic variant allele frequency (VAF) of approximately 10%. However, targeted NGS panels with a minimum read depth of approximately 350× can detect variants with a VAF limit of detection of 4% in some cases. This is dependent on the variant type and may be confounded by variable read depth in some regions. Since *NF2*-SWN has such a high frequency of mosaic disease, if no variant is found by standard targeted sequencing, a subsequent higher read-depth test, optimised to detect mosaic variants, may be used to detect very low-level variants that are present in blood, but which may have been missed on standard testing. This can detect mosaic variants below 1% VAF in some cases, particularly when tumour DNA is available for tumour-guided analysis. This method enables variant assessment using a single tumour and a blood sample. However, extremely low levels of mosaicism may still not be detectable in blood. These variants may only be identified by detection of an identical variant in two anatomically distinct tumours. The use of two separate tumours has been shown to increase detection of mosaic *NF2* variants when the variant is not detectable in blood, even on a high read-depth assay. However, multiple tumours are not available for the majority of people, which accounts for a significant proportion of unconfirmed mosaic disease. In Manchester data, 43/179 (24%) people tested for mosaic variant fraction by NGS had a low-level variant detected in blood at below 4% VAF, and four of these were below 1%. In 91/179 (51%) no variant was detected in blood.

Copy number variants (CNVs) can be detected within targeted sequencing data using bioinformatic tools such as, DECoN [89], and any findings are confirmed by orthogonal techniques, such as multiplex ligation-dependent probe amplification (MLPA) using probe mixes from MRC-Holland, or by droplet digital PCR. These techniques may not detect certain CNVs, such as those involving repetitive sequences, or which are not captured in target regions. MLPA also has a relatively low sensitivity for detection of mosaic variants, which need to be greater than 30%. This is significant, particularly for *NF2*-SWN, due to the high proportion of causative CNVs [26] and the high incidence of mosaicism [29] as it means that routine clinical genetic testing may fail to detect a significant number of low-level mosaic CNVs.

Deep-intronic splice variants account for around 1–5% of all pathogenic variants and have been identified in *NF2*-SWN and *SMARCB1*-SWN [59, 60, 82, 90–92]. These are not normally covered by standard targeted sequencing panels. Even panels that aim to include the entire gene sequence of the major schwannomatosis genes may not provide adequate coverage in these regions. When a potential splice variant is identified that is not located in the canonical ± 1,2, splice sites, or within the splice acceptor (− 20 to + 1) or splice donor (-3 to + 6) regions, and it is not clear from in silico predictions, what effect the variant will have on the resulting transcript, it is challenging to interpret whether the variant is likely to be pathogenic. Some splice variants that are predicted to cause skipping of a single exon that maintains the reading frame, may in fact cause skipping of multiple exons, part of an exon, or aberrant inclusion of intronic sequences, which may disrupt the reading frame. This can significantly affect the overall likelihood of pathogenicity and/or the associated disease severity. Standard cDNA analysis using RT-PCR and short-read sequencing can detect many of these changes [60, 62, 82, 83, 93], but this method is only semi-quantitative and may not detect isoform-specific changes or enable assessment of multiple splice products, therefore it may be helpful to include quantitative RNA-sequencing in these cases.

Only variants classified as pathogenic or likely pathogenic according to American College of Medical Genetics and Genomics (ACMG) variant interpretation framework [94] should be reported back to patients, but other variants classified as variants of uncertain significance, benign, or likely benign can be stored in case further evidence is acquired that can be used to re-classify the variant at a later date. Ongoing efforts to define the clinical significance of variants within each of the known schwannomatosis genes are facilitated by the National Institutes of Health-funded Clinical Genome resource (ClinGen: https://clinicalgenome.org) through a variant curation expert panel (VCEP). The VCEP aims to provide gene-specific guidance for the application of the ACMG framework to schwannomatosis-related variants.

There are still many cases that have been tested for all of the known causative genes and no variant has been found [60, 70, 95]. These are currently classified as schwannomatosis not elsewhere classified (SWN-NEC) [1]. A proportion of these genetically unconfirmed cases may be due to mosaic disease, particularly for *NF2*-SWN [29]. However, there remains the possibility that there are other schwannomatosis genes still to be discovered. These genes, as well as additional variant types that are not readily detectable through standard testing, may be found as the use of newer technologies, such as long-read sequencing, becomes more common.

## Data Availability

No datasets were generated or analysed during the current study.
